# Usefulness of patch tests in drug adverse reaction induced by tetrazepam and anti-inflammatory drugs

**DOI:** 10.1186/2045-7022-4-S3-P106

**Published:** 2014-07-18

**Authors:** Haudrey Assier, Cynthia Haddad, Jean-Claude Roujeau, Pierre Wolkenstein, Olivier Chosidow, Laurence Valeyrie-Allanore

**Affiliations:** 1 Department of dermatology, Henri Mondor Hospital, France; 2 Henri Mondor Hospital, Department of Dermatology, France

## 

Skin testing with the suspected drugs may be helpful in determining the cause of a cutaneous adverse reaction (CADR). Tetrazepam (T) is a muscle relaxant of the benzodiazepine group frequently used in rheumatology in association with anti-inflammatory drugs (AIDs). We propose to determine the usefulness of patch testing in this context. Patients and methods A retrospective study was conducted including all patients referred between 2000 and 2010 for patch testing after a CADR occurring after concomitant administration of T and AIDs. Patch test were performed at least 6 weeks after healing with T and AIDs diluted at 10 % in petrolatum in a commercial form (Chemotechnique Laboratory Diagnostics, Malmö, Sweden) or crushed and diluted in petroleum. All patients were contacted by letter in 2010 and data concerning T or AID rechallenge, after the patch testing, were collected. T patch testing was also performed on 10 controls, ie individuals who already took T but did not present any CADR.

## Results

15 subjects with a past history of CADR were referred for patch testing with both T and AIDs possible / probable culprit drugs. Among this patients, CADR included Stevens Johnson/toxic epidermal necrolysis (5), maculopapular rash (7), Drug Reaction with Eosinophilia and Systemic Symptoms (1) and Acute Generalized Exanthematous Pustulosis (2). AIDs included ketoprofene (3), diclofenac (2), rofecoxib (2), piroxicam (5), naproxène (1), prednisone (1), cortivazol (1). Patch tests were positive for T and negative for AIDs in 80 % of cases (12). Both patch tests were negative in 20% (3). All the 10 control subjects had negative patch test to T. Nine subjects answered to our letter, none of them has been rechallenged with either T or AIDs, whatever the results of the tests. Comments Positive T patch tests were obtained in 80% of cases whatever the CARDs. This was much higher than the usual 30-50% rate observed in the literature with miscellaneous drugs. All the T exposed control subjects were negative as were 100 non exposed controls reported in literature. However, its predictive value can not be determined in absence of rechallenge after negative patch test. T CADR may be systematically suspected even in case of concomitant AIDs intake. Readministration of AID could be allowed in non severe CADR with negative AID patch testing and positive one for T.

